# Supplementary material for: Transcriptional Regulation of Autophagy Genes via Stage-Specific Activation of CEBPB and PPARG during Adipogenesis: A Systematic Study Using Public Gene Expression and Transcription Factor Binding Datasets
Source: Cells. 2019 Oct 25;8(11):1321. doi: 10.3390/cells8111321 (PMC6912425; doi:10.3390/cells8111321)
Supplement: Supplementary file 1 [file cells-08-01321-s001.pdf]

Supplementary Materials: Transcriptional regulation of autophagy genes via stage-specific activation of CEBPB and PPARG during adipogenesis: a systematic study using public gene expression and transcription factor binding datasets

Mahmoud Ahmed, Trang Huyen Lai, Jin Seok Hwang, Sahib Zada, Trang Minh Pham and Deok Ryong Kim *,

List of Tables

S1 Percent of gene expression variance explained by the stage of differentiation 2
S2 Differential expression of gene markers 3
S3 Autophagy and lipid metabolism gene ontology terms enrichment in differentiating adipocytes 4
S4 Significant peaks of adipogenic factors on autophagy genes 5
S5 Significant peaks of adipogenic factors on autophagy transcription factor genes 6
S6 Autophagy and lipid metabolism gene ontology terms enrichment in *Cebpb* and *Pparg*-knockdown differentiating adipocytes 7
S7 Peaks of adipogenic factors on adipogenic transcription factor genes 8
S8 Datasets of RNA and ChIP Seq 9
S9 ChIP antibodies for transcription factors, co-factors and histone markers. 10
S10 Datasets of ChIP Seq in different tissues 11
S11 Datasets of RNA Seq and microarrays of adipocytes with *Cebpb* or *Pparg*-knockdown . . 12

List of Figures

S1 Differential gene expression in early and late differentiating adipocytes. 13
S2 Differential peak binding of adipogenic transcription factors on autophagy genes. . . . 14
S3 Gene markers of differentiation, lipogenesis and autophagy. 15
S4 Co-expression of adipogenic transcription factors genes with autophagy genes 16
S5 Tissue specific binding of adipogenic transcription factors 17
S6 Adipogenic transcription factors correlations with co-factors and histone markers on autophagy genes. 18
S7 Correlation in occupancy between adipogenic transcription factors and histone markers on autophagy genes at different genomic locations. 19
S8 Differential gene expression in *Cebpb* or *Pparg*-knockdown differentiating adipocytes. . 20

Table S1. Percent of gene expression variance explained by the stage of differentiation. Dim, dimension; TF, transcription factor.

Category	Dim 1	Dim 2
All Genes	34	17
Autophagy Genes	32	21
Adipogenic TF	62	23
Autophagy TF	76	11

Table S2. Differential expression of gene markers. FC, fold-change; SE, standard error; FDR, false discovery rate.

Category	Gene	Early vs Non			Late vs Non			Late vs Early		
		FC	SE	FDR	FC	SE	FDR	FC	SE	FDR
Adipogenesis	Cebpa	2.55	0.38	< 0.2	3.8	0.38	< 0.2	1.25	0.35	< 0.2
	Cebpb	1.5	0.19	< 0.2	0.2	0.19	0.39	-1.3	0.18	< 0.2
	Pparg	1.55	0.25	< 0.2	2.76	0.25	< 0.2	1.21	0.23	< 0.2
Autophagy	Becn1	0.31	0.08	< 0.2	0.44	0.09	< 0.2	0.13	0.08	< 0.2
	Map1lc3b	-0.72	0.17	< 0.2	-0.27	0.17	< 0.2	0.45	0.15	< 0.2
	Sqstm1	-1.02	0.15	< 0.2	-0.11	0.15	0.57	0.91	0.14	< 0.2
Lipogenesis	Acly	0.48	0.2	< 0.2	0.62	0.2	< 0.2	0.14	0.19	0.53
	Fasn	1.22	0.27	< 0.2	1.98	0.28	< 0.2	0.76	0.25	< 0.2
	Lpl	0.01	0.28	0.98	2.62	0.29	< 0.2	2.61	0.27	< 0.2

Table S3. Autophagy and lipid metabolism gene ontology terms enrichment in differentiating adipocytes. GO, gene ontology; Ratio, ratio of differentially expressed genes in terms.

Category	GO Term (ID)	Early vs Non		Late vs Non		Late vs Early	
		Ratio	P-value	Ratio	P-value	Ratio	P-value
Autophagy	negative regulation of macroautophagy (GO:0016242)	0.75	0.02				
	positive regulation of autophagosome maturation (GO:1901098)			1	0.01		
	positive regulation of autophagy of mitochondrion in response to mitochondrial depolarization (GO:1904925)					1	0.02
Lipid Metabolism	ether lipid biosynthetic process (GO:0008611)			1	0.02		
	glycerophospholipid biosynthetic process (GO:0046474)			1	0.05		
	glycerophospholipid metabolic process (GO:0006650)					1	< 0.01
	glycosphingolipid metabolic process (GO:0006687)					1	0.02
	lipid biosynthetic process (GO:0008610)			0.68	0.05		
	lipid catabolic process (GO:0016042)	0.52	0.01	0.69	< 0.01	0.6	< 0.01
	lipid droplet (GO:0005811)			0.6	0.02	0.68	< 0.01
	lipid glycosylation (GO:0030259)					0.8	0.03
	lipid homeostasis (GO:0055088)			0.69	< 0.01	0.69	< 0.01
	lipid metabolic process (GO:0006629)	0.47	< 0.01	0.63	< 0.01	0.6	< 0.01
	lipid storage (GO:0019915)	0.65	< 0.01	0.69	0.02	0.88	< 0.01
	lipid transport (GO:0006869)	0.51	0.01	0.65	< 0.01	0.59	< 0.01
	negative regulation of lipid catabolic process (GO:0050995)			0.75	0.05	0.83	0.01
	negative regulation of lipid storage (GO:0010888)	0.83	0.04				
	phospholipid catabolic process (GO:0009395)			0.79	0.02		
	phospholipid efflux (GO:0033700)			0.8	0.03	0.8	0.03
	phospholipid homeostasis (GO:0055091)			0.82	0.02	0.82	0.02
	phospholipid metabolic process (GO:0006644)					0.71	0.01
	phospholipid scrambling (GO:0017121)	1	< 0.01				
	phospholipid translocation (GO:0045332)	0.62	0.02				
	phospholipid transport (GO:0015914)					0.68	0.01
	positive regulation of lipid biosynthetic process (GO:0046889)			0.87	< 0.01	0.8	< 0.01
	positive regulation of lipid catabolic process (GO:0050996)					1	< 0.01
	positive regulation of lipid storage (GO:0010884)					0.8	0.03
	regulation of lipid biosynthetic process (GO:0046890)	0.83	0.04				

Table S4. Significant peaks of adipogenic factors on autophagy genes. N, number of peaks; Range, range of fold-change; Ave, average fold-change; SD, standard deviation.

Category	Factor	Gene	Early vs Non				Late vs Non				Late vs Early			
			(N)	Range	Ave	SD	(N)	Range	Ave	SD	(N)	Range	Ave	SD
Factor	CEBPB	Becn1	1	-1.13	-1.13						1	1.71	1.71	
		Map1lc3b	1	-1.34	-1.34						1	1.7	1.7	
	PPARG	Sqstm1									1	-2.75	-2.75	
Cofactor	MED1	Becn1									1	1.12	1.12	
		Map1lc3b									1	1.13	1.13	
		Sqstm1	2	-0.85/0.77	-0.04	1.14					2	-0.5/1.34	0.42	1.3
		Ulk1					1	1.27	1.27		1	0.99	0.99	
	RXRG	Sqstm1									1	1.14	1.14	
Histone Marker	H3K27ac	Becn1					1	2.1	2.1		1	2.37	2.37	
		Map1lc3a									1	2.49	2.49	
		Map1lc3b	1	1.13	1.13		2	1.7/1.9	1.8	0.14	2	2.11/2.35	2.23	0.17
		Sqstm1	2	-1.6/1.52	-0.04	2.21					2	-1.51/3.08	0.79	3.25
		Ulk1									1	2.88	2.88	

Table S5. Significant peaks of adipogenic factors on autophagy transcription factor genes. N, number of peaks; Range, range of fold-change; Ave, average fold-change; SD, standard deviation.

Category	Factor	Gene	Early vs Non				Late vs Non				Late vs Early			
			(N)	Range	Ave	SD	(N)	Range	Ave	SD	(N)	Range	Ave	SD
Factor	CEBPB	Foxo1	1	-0.86	-0.86									
		Xbp1	1	1.1	1.1		1	1.43	1.43		1	1.45	1.45	
	PPARG	Tfeb					1	1.4	1.4					
Cofactor	EP300	Xbp1	1	1.15	1.15		1	2.41	2.41		1	1.26	1.26	
	MED1	Foxo1	1	2.65	2.65		8	0.89/2.81	1.62	0.57	6	0.89/1.36	1.05	0.21
		Tfeb					3	1.84/2.92	2.4	0.54	4	1.41/1.72	1.55	0.13
		Xbp1	3	1.97/2.79	2.4	0.41	6	1.22/2.98	2.26	0.68	2	0.58/0.69	0.63	0.08
		Zkscan3	1	2.87	2.87						2	-1.56/0.79	-0.39	1.66
	RXRG	Foxo1									2	0.78/1.26	1.02	0.34
		Tfeb									4	0.97/2	1.45	0.44
		Xbp1									1	-0.73	-0.73	
Histone Marker	H3K27ac	Foxo1									2	2.17/3.15	2.66	0.69
		Xbp1									1	2.77	2.77	
	H3K4me3	Foxo1	1	-1.4	-1.4									
		Xbp1	1	0.53	0.53									

Table S6. Autophagy and lipid metabolism gene ontology terms enrichment in *Cebpb*- and *Pparg*-knockdown differentiating adipocytes. GO, gene ontology; KD, knockdown; h, hour; d, day.

Category	GO Term (ID)	Cebpb KD	Pparg KD
Autophagy	autophagic cell death(GO:0048102)		5d
	autophagosome assembly(GO:0000045)		2d
	autophagosome maturation(GO:0097352)	0h	2d
	autophagy of peroxisome(GO:0030242)		4d
	autophagy(GO:0006914)		2d
	positive regulation of autophagy(GO:0010508)		2d
	positive regulation of macroautophagy(GO:0016239)	0h/4h	
Lipid Metabolism	C-terminal protein lipidation(GO:0006501)		5d
	cellular lipid catabolic process(GO:0044242)		2d/5d
	cellular lipid metabolic process(GO:0044255)		4d
	cellular sphingolipid homeostasis(GO:0090156)		0d
	ether lipid biosynthetic process(GO:0008611)		4d/5d
	ether lipid metabolic process(GO:0046485)	4h	
	glycolipid binding(GO:0051861)		6d
	glycolipid biosynthetic process(GO:0009247)	4h	
	lipid binding(GO:0008289)		2d/4d/5d
	lipid biosynthetic process(GO:0008610)		4d/5d/6d
	lipid catabolic process(GO:0016042)		4d
	lipid droplet organization(GO:0034389)		2d
	lipid droplet(GO:0005811)	0h/4h	4d/5d/6d
	lipid homeostasis(GO:0055088)		0d/5d/6d
	lipid metabolic process(GO:0006629)	0h/4h	0d/2d/4d/5d/6d
	lipid storage(GO:0019915)	4h	2d/4d/5d/6d
	lipid transport(GO:0006869)	4h	4d/5d/6d
	lipid transporter activity(GO:0005319)	0h	
	negative regulation of lipid transport(GO:0032369)		0d
	phospholipid metabolic process(GO:0006644)	4h	
	phospholipid scrambling(GO:0017121)		2d
	phospholipid transport(GO:0015914)	0h/4h	
	phospholipid transporter activity(GO:0005548)		2d
	positive regulation of lipid biosynthetic process(GO:0046889)		0d
	positive regulation of phospholipid efflux(GO:1902995)		0d
	protein delipidation(GO:0051697)		2d
	protein lipidation(GO:0006497)		5d
	regulation of lipid storage(GO:0010883)		4d/6d
	sphingolipid mediated signaling pathway(GO:0090520)	4h	

Table S7. Peaks of adipogenic factors on adipogenic transcription factor genes. N, number of peaks; Range, range of fold-change; Ave, average fold-change; SD, standard deviation.

Factor	Gene	Early vs Non				Late vs Non				Late vs Early			
		(N)	Range	Ave	SD	(N)	Range	Ave	SD	(N)	Range	Ave	SD
CEBPB	Cebpb	1	-0.35	-0.35		1	1.22	1.22		1	1.57	1.57	
	Pparg	6	-0.08/0.88	0.5	0.37	6	-0.71/0.21	-0.22	0.42	6	-1.35/0.3	-0.71	0.65
PPARG	Cebpb	1	0.38	0.38		1	0.56	0.56		1	0.18	0.18	
	Pparg	1	-0.16	-0.16		1	-0.48	-0.48		1	-0.31	-0.31	

Table S8. Datasets of RNA and ChIP Seq. N, the number of samples; PIMD, PubMed ID; Ref., reference.

Type	Study	(N)	Factor	Time (hr)	Stage	PMID	Ref
ChIP Seq	SRP100871	20	H3K27ac/H3K4me1/H3K4me2/MED1/EP300	0/4/48/96/168	non/early/late	28475875	[1]
	SRP000630	18	PPARG/RXRG/POLR2A	0/24/48/72/96/144	non/early/late	18981474	[2]
	SRP002337	10	H3K4me3/H3K4me2/H3K4me1/H3K27ac/PPARG	-48/0/48/168	non/early/late	20887899	[3]
	SRP006001	9	CEBPB/RXRG/PPARG/POLR2A	0/2/4/48/144	non/early/late	21427703	[4]
	SRP041249	9	RXRG/MED1/EP300/H3K27ac/H3K4me1/H3K4me2	4	early	24857652	[5]
	SRP078506	6	H3K4me3	0/4/48	non/early	27899593	[6]
	SRP028367	5	PPARG/MED1/POLR2A	168	late	23885096	[7]
	SRP064188	5	H3K9me3/POLR2A	0/144	non/late	26590716	[8]
	SRP041129	4	MED1/EP300/CEBPB	NA	late	24788520	[9]
	SRP043216	3	H3K27ac/H3K4me1/H3K4me3	NA	non	25503565	[10]
	SRP002507	2	CEBPB	0/6	non/early	20478996	[11]
	SRP080809	2	CEBPB	NA	non	28107648	[12]
	SRP009613	1	PPARG	NA	non	24315104	[13]
	SRP016054	1	H3K4me3	NA	non	23178591	[14]
RNA Seq	SRP045772	12		0/24/48	non/early	26220403	[15]
	SRP100871	10		4/0/24/48/168	early/non/late	28475875	[1]
	SRP100171	8		0/48/144/192	non/early/late	29317436	[16]
	SRP029985	6		0/168	non/late	24912735	[17]
	SRP051775	6		168	late	25596527	[18]
	SRP102079	6		0/2/4	non/early	29748257	[19]
	SRP041765	4		0/4	non/early	24857666	[20]
	SRP010905	3		192	late	24095730	[21]
	SRP033778	3		0/48/240	non/early/late	25412662	[22]
	SRP066991	3		0/48/168	non/early/late	27923061	[23]
	SRP092793	3		240	late	28009298	[24]
	SRP078506	2		0/4	non/early	27899593	[6]

Table S9. ChIP antibodies for transcription factors, co-factors and histone markers. Ref., reference.

Factor	Antibody	Study	Ref.
CEBPB	sc-150, Santa Cruz	SRP041129, SRP080809, SRP002507, SRP006001	[4,9,11,12]
EP300	sc-585, Santa Cruz; sc-584, Santa Cruz	SRP041129, SRP041249, SRP100871	[1,5,9]
H3K27ac	ab4729, Abcam; 39133, Active Motif	SRP041249, SRP043216, SRP100871, SRP002337	[1,3,5,10]
H3K4me1	ab8895, Abcam	SRP041249, SRP043216, SRP100871, SRP002337	[1,3,5,10]
H3K4me2	9726, Cell Signalling; ab7766, Abcam	SRP041249, SRP100871, SRP002337	[1,3,5]
H3K4me3	07-473, Millipore; 8580, Abcam; ab8580, Abcam	SRP016054, SRP043216, SRP078506, SRP002337	[3,6,10,14]
H3K9me3	2F3, In Lab	SRP064188	[8]
MED1	sc-8998, Santa Cruz; A300-793A, Bethyl Laboratories	SRP028367, SRP041129, SRP041249, SRP100871	[1,5,7,9]
POLR2A	AC-055-100, Diagenode; C13B9, In Lab; AC-0555-100, Diagenode	SRP028367, SRP064188, SRP000630, SRP006001	[2,4,7,8]
PPARG	NA; sc-7196, Santa Cruz; sc-7196, Santa Cruz; 2443, Cell Signalling	SRP009613, SRP028367, SRP000630, SRP002337, SRP006001	[2–4,7,13]
RXRG	sc-774, Santa Cruz	SRP041249, SRP000630, SRP006001	[2,4,5]

Table S10. Datasets of ChIP Seq in different tissues. N, the number of samples; PIMD, PubMed ID; Ref., reference.

Tissue	Study	(N)	Factor	PMID	Ref
Adipocyte	GSE83757	2	CEBPB	28812581	[25]
	GSE67586	2	PPARG	25948511	[26]
Fibroblast	GSE44942	2	CEBPB	23590861	[27]
	GSE41937	2	PPARG	23326641	[28]
Macrophage	GSE69099	1	CEBPB	26923725	[29]
	GSE63696	1	PPARG	25574839	[30]
	GSE92606	1	PPARG	28240605	[31]

Table S11. Datasets of RNA Seq and microarrays of adipocytes with *Cebpb* or *Pparg*-knockdown. N, the number of samples; PIMD, PubMed ID; Ref., reference.

Study	(N)	KD Gene	Time	PMID	Ref.
GSE57415	8	Cebpb	0/4h	24857666	[20]
GSE12929	18	Pparg	0/2d/5d	19300518	[32]

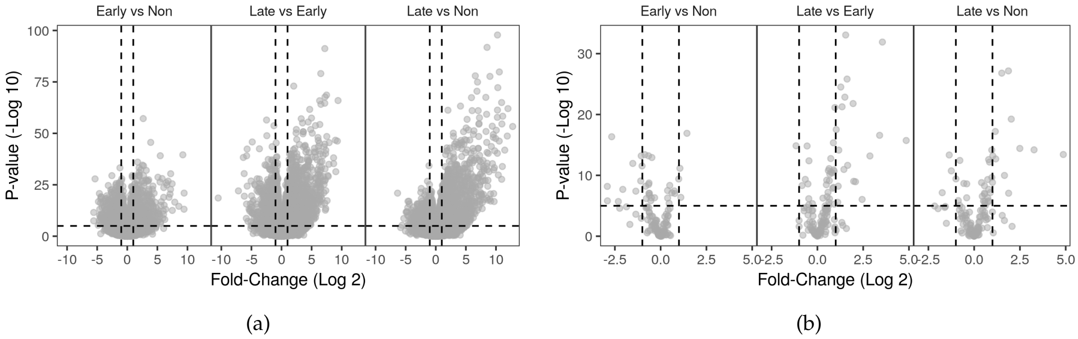

Figure S1. Differential gene expression in early and late differentiating adipocytes. Read counts of (a) 15786 coding genes (b) 158 autophagy genes from RNA-Seq samples (n = 66) were used to perform differential expression analysis between early/late differentiation stage and non-differentiated samples. Points represent individual gene fold-change and p-value on the log scales.

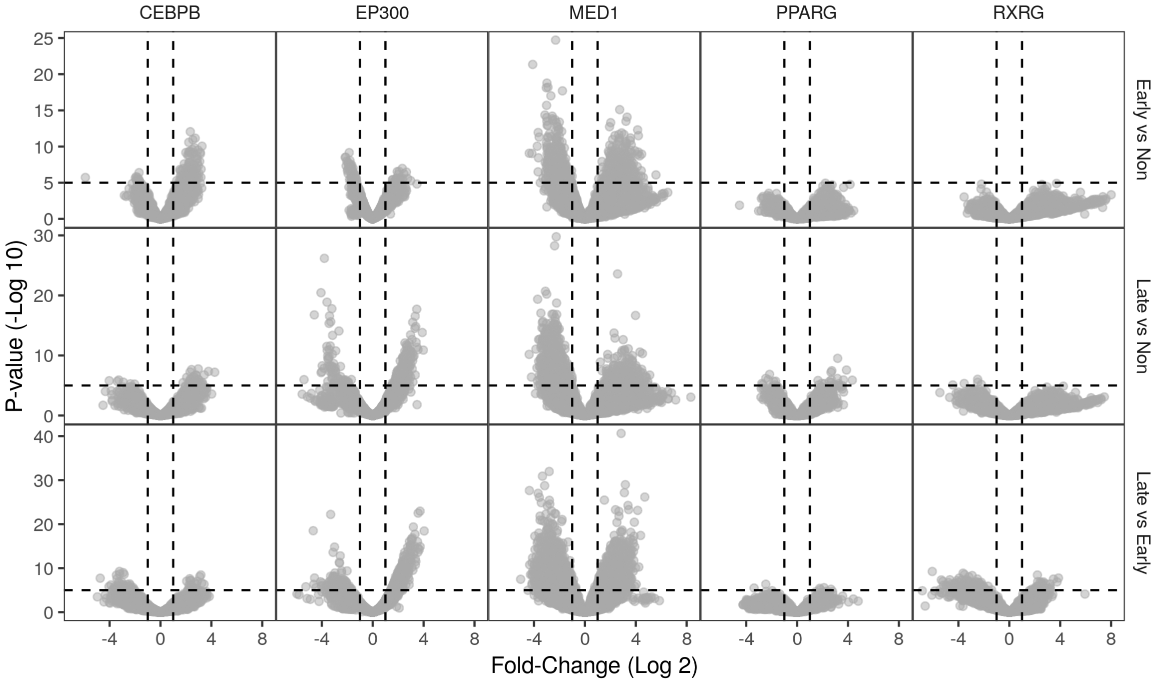

(a)

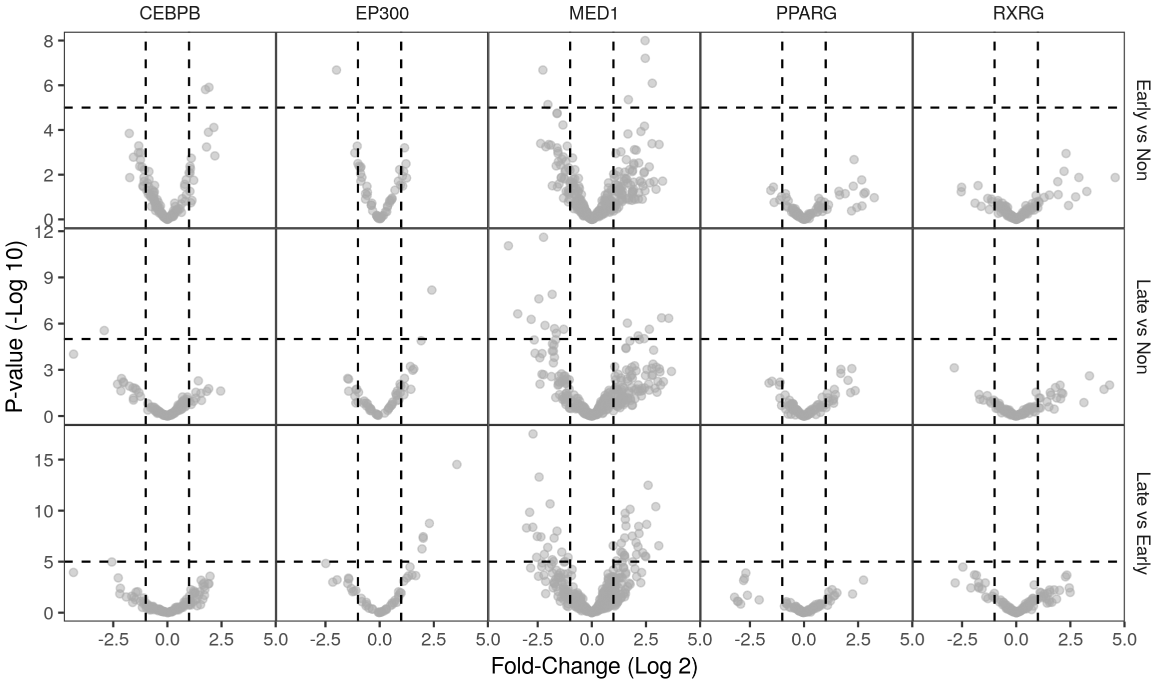

(b)

Figure S2. Differential peak binding of adipogenic transcription factors on autophagy genes. Reads count in peaks in (a) coding genes and (b) 158 autophagy genes from ChIP-Seq samples (n = 50) were used to perform differential peak binding analysis between early/late differentiation stage and non-differentiated samples. Points represent individual gene fold-change and p-value on log scales.

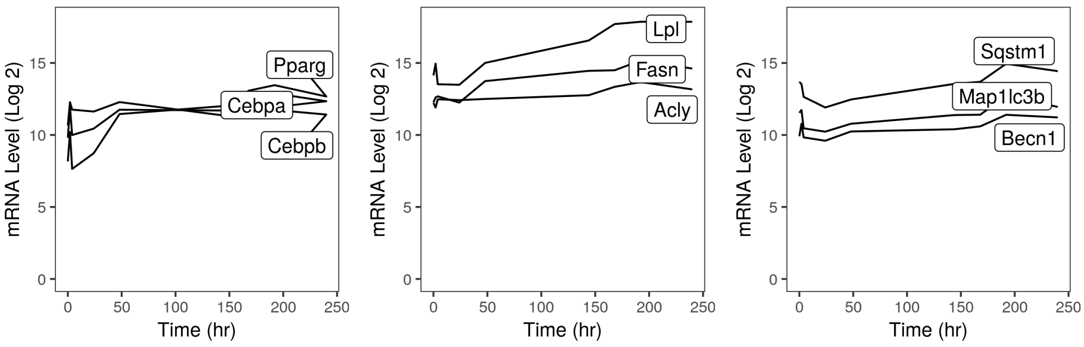

(a) (b) (c)

Figure S3. Gene markers of differentiation, lipogenesis and autophagy. Gene counts from RNA-Seq samples (n = 66) of MDI-induced 3T3-L1 at multiple differentiation time points were used to determine the mRNA expression of genes. The $\log_2$ mRNA level of (a) three adipogenic markers *Cebpa*, *Cebpb* and *Pparg*, (b) three lipogenesis markers *Lpl*, *Fasn* and *Acly* and (c) three autophagy markers *Map1lc3b*, *Sqstm1* and *Becn1* are shown as lines over time.

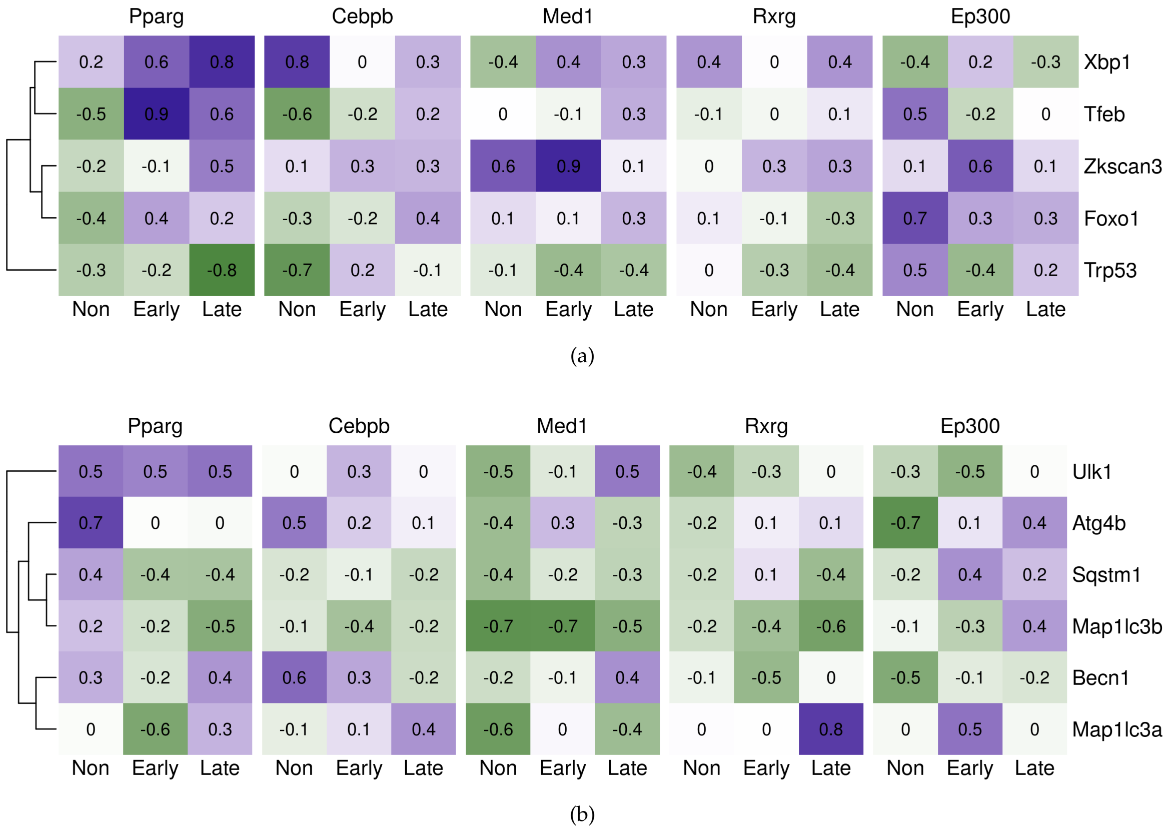

Figure S4. Co-expression of adipogenic transcription factors genes with autophagy genes. Read counts of genes from RNA-Seq samples (n = 66) was used to measure the gene expression. The expression of five adipogenic transcription factor genes was used to calculate the co-expression (a) with key autophagy genes and (b) with autophagy transcription factor genes. The transformed reads count for each pair of genes were used to calculate Pearson's Correlation Coefficients (PCC) for the pair. The PCC values between -1 (green) and 1 (blue).

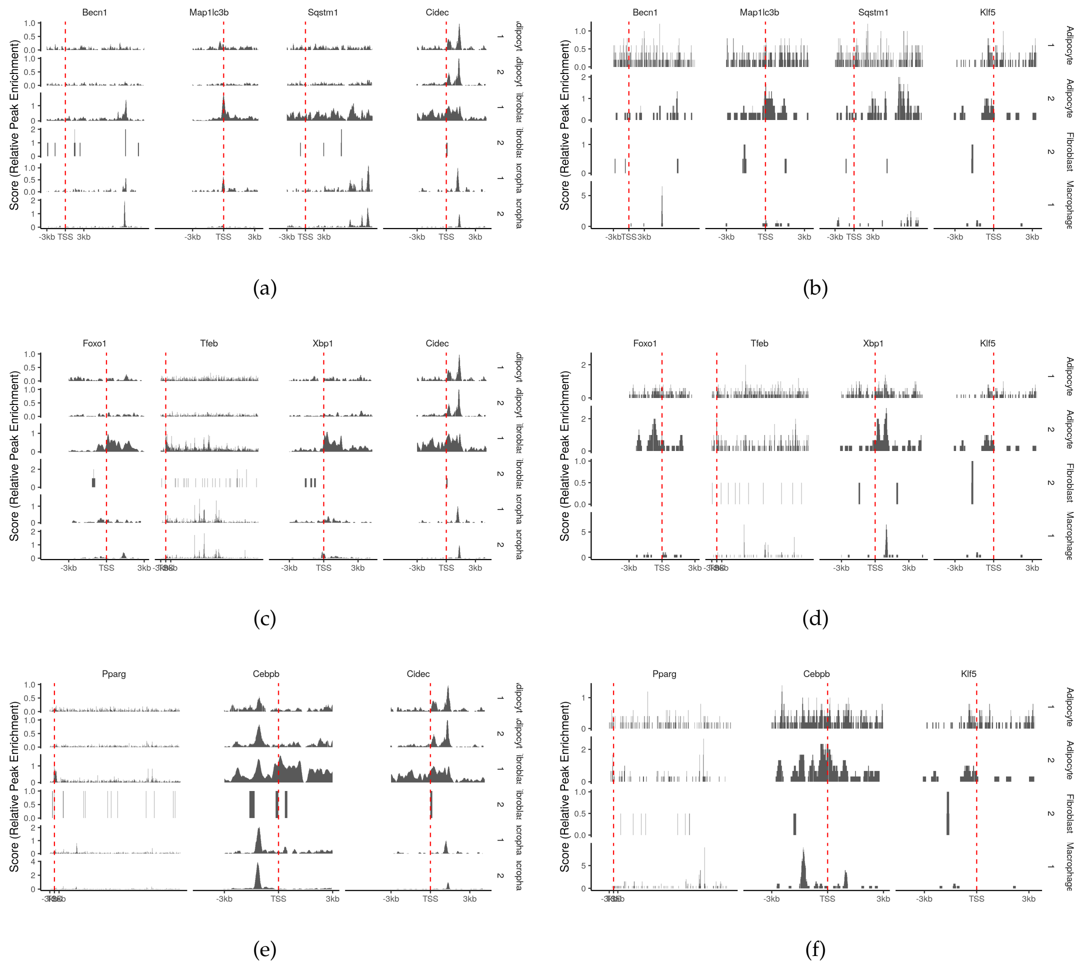

Figure S5. Tissue specific binding of adipogenic transcription factors. Signal tracks from (a, c & e) PPARG and (b, d & f) CEBPB ChIP-Seq data of three different tissue types; Adipocyte, Fibroblast and Macrophage were obtained from Cistrome Data Browser. The signal score around the promoter regions of (a & b) key autophagy genes, (c & d) autophagy transcription factor genes and (e & f) adipogenic transcription factor genes were visualized. *Cidec* and *klf5* were used as positive control genes. Promoters are $\pm 3kb$ around the transcription start sites.

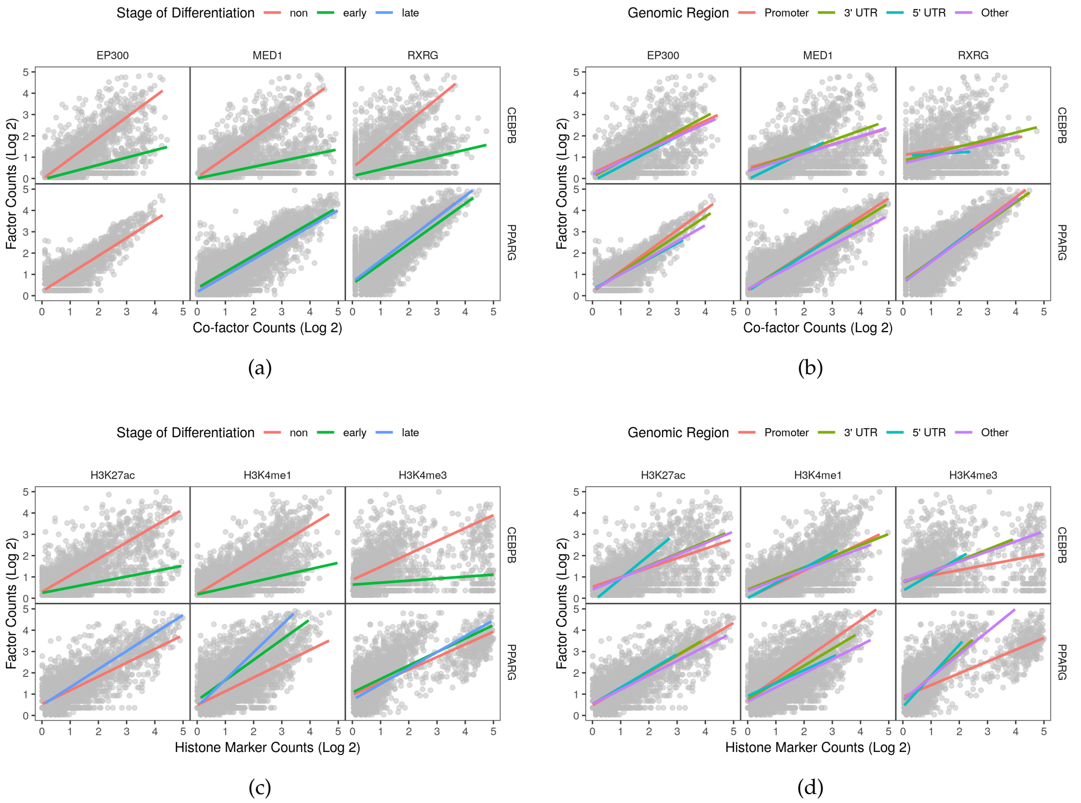

Figure S6. Adipogenic transcription factors correlations with co-factors and histone markers on autophagy genes. The total number of reads in peaks of each ChIP-Seq sample (n = 75) was used to represent the factor/histone marker occupancy. The occupancy of two adipogenic transcription factors CEBPB and PPARG was plotted against that of (a) & (c) three co-factors and (b) & (d) histone markers. Samples were categorized by differentiation stage; non, early or late in that order in (a) & (b). Peaks were categorized by the genomic location into Promoter, 3' UTR, 5' UTR and other in that order in (c) & (d).

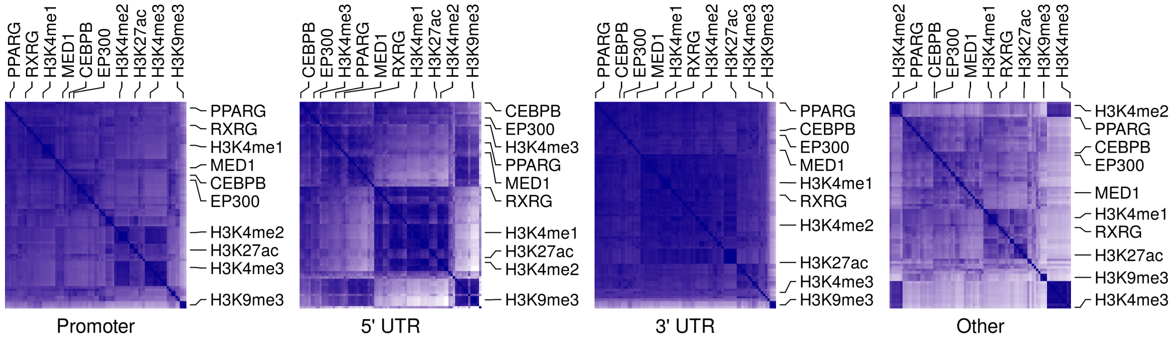

Figure S7. Correlation in occupancy between adipogenic transcription factors and histone markers on autophagy genes at different genomic locations. The total number of reads in peaks of each ChIP-Seq sample was used to represent the factor/histone marker occupancy. Pearson's Correlation Coefficient (PCC) was calculated for each pair of samples. The PCC value is between 0 (white) and 1 (blue). Peaks were categorized by the genomic location into Promoter, 3′ UTR, 5′ UTR and other in that order.

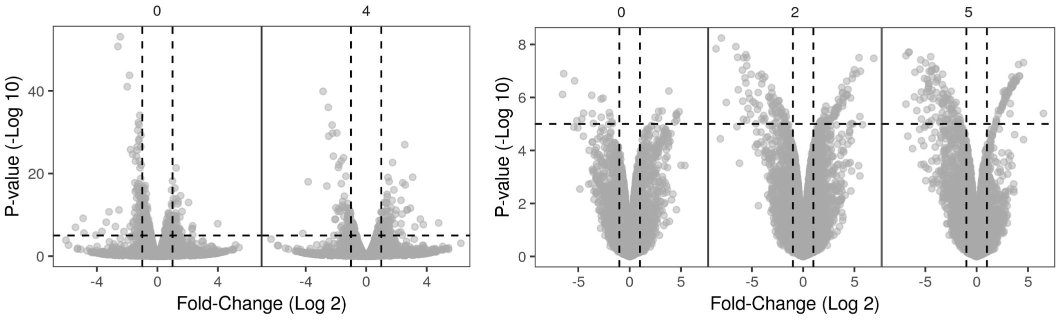

(a) (b)

Figure S8. Differential gene expression in *Cebpb* or *Pparg*-knockdown differentiating adipocytes. Read counts of all coding genes from (a) RNA-Seq samples (n = 8) of *Cebpb*-knockdown adipocytes at 0 and 4 hours (b) microarray samples (n = 18) of *Pparg*-knockdown adipocytes at 0, 2 and 5 days were used to perform differential expression analysis between knockdown vs control samples of differentiation adipocytes at different time points. Points represent individual gene fold-change and p-value on log scales.

Supplementary References

1. Siersbæk, R.; Madsen, J.G.S.; Javierre, B.M.; Nielsen, R.; Bagge, E.K.; Cairns, J.; Wingett, S.W.; Traynor, S.; Spivakov, M.; Fraser, P.; Mandrup, S. Dynamic Rewiring of Promoter-Anchored Chromatin Loops during Adipocyte Differentiation. *Molecular Cell* **2017**, *66*, 420–435. doi:10.1016/j.molcel.2017.04.010.
2. Nielsen, R.; Pedersen, T.A.; Hagenbeek, D.; Moulos, P.; Siersbaek, R.; Megens, E.; Denissov, S.; Børgesen, M.; Francoijs, K.J.; Mandrup, S.; Stunnenberg, H.G. Genome-wide profiling of PPARgamma:RXR and RNA polymerase II occupancy reveals temporal activation of distinct metabolic pathways and changes in RXR dimer composition during adipogenesis. *Genes & development* **2008**, *22*, 2953–67. doi:10.1101/gad.501108.
3. Mikkelsen, T.S.; Xu, Z.; Zhang, X.; Wang, L.; Gimble, J.M.; Lander, E.S.; Rosen, E.D. Comparative epigenomic analysis of murine and human adipogenesis. *Cell* **2010**, *143*, 156–169. doi:10.1016/j.cell.2010.09.006.
4. Siersbfk, R.; Nielsen, R.; John, S.; Sung, M.H.; Baek, S.; Loft, A.; Hager, G.L.; Mandrup, S. Extensive chromatin remodelling and establishment of transcription factor hotspots during early adipogenesis. *EMBO Journal* **2011**, *30*, 1459–1472. doi:10.1038/emboj.2011.65.
5. Siersbæk, R.; Rabiee, A.; Nielsen, R.; Sidoli, S.; Traynor, S.; Loft, A.; Poulsen, L.L.C.; Rogowska-Wrzesinska, A.; Jensen, O.N.; Mandrup, S. Transcription factor cooperativity in early adipogenic hotspots and super-enhancers. *Cell Reports* **2014**, *7*, 1443–1455. doi:10.1016/j.celrep.2014.04.042.
6. Brier, A.S.B.; Loft, A.; Madsen, J.G.; Rosengren, T.; Nielsen, R.; Schmidt, S.F.; Liu, Z.; Yan, Q.; Gronemeyer, H.; Mandrup, S. The KDM5 family is required for activation of pro-proliferative cell cycle genes during adipocyte differentiation. *Nucleic Acids Research* **2017**, *45*, 1743–1759. doi:10.1093/nar/gkw1156.
7. Haakonsson, A.K.; Stahl Madsen, M.; Nielsen, R.; Sandelin, A.; Mandrup, S. Acute Genome-Wide Effects of Rosiglitazone on PPARγ Transcriptional Networks in Adipocytes. *Molecular Endocrinology* **2013**, *27*, 1536–1549. doi:10.1210/me.2013-1080.
8. Matsumura, Y.; Nakaki, R.; Inagaki, T.; Yoshida, A.; Kano, Y.; Kimura, H.; Tanaka, T.; Tsutsumi, S.; Nakao, M.; Doi, T.; Fukami, K.; Osborne, T.F.; Kodama, T.; Aburatani, H.; Sakai, J. H3K4/H3K9me3 Bivalent Chromatin Domains Targeted by Lineage-Specific DNA Methylation Pauses Adipocyte Differentiation. *Molecular Cell* **2015**, *60*, 584–596. doi:10.1016/j.molcel.2015.10.025.
9. Step, S.E.; Lim, H.W.; Marinis, J.M.; Prokesch, A.; Steger, D.J.; You, S.H.; Won, K.J.; Lazar, M.A. Anti-diabetic rosiglitazone remodels the adipocyte transcriptome by redistributing transcription to PPARγ-driven enhancers. *Genes and Development* **2014**, *28*, 1018–1028. doi:10.1101/gad.237628.114.
10. Kang, S.; Tsai, L.T.; Zhou, Y.; Evertts, A.; Xu, S.; Griffin, M.J.; Issner, R.; Whitton, H.J.; Garcia, B.A.; Epstein, C.B.; Mikkelsen, T.S.; Rosen, E.D. Identification of nuclear hormone receptor pathways causing insulin resistance by transcriptional and epigenomic analysis. *Nature Cell Biology* **2015**, *17*, 44–56. doi:10.1038/ncb3080.
11. Steger, D.J.; Grant, G.R.; Schupp, M.; Tomaru, T.; Lefterova, M.I.; Schug, J.; Manduchi, E.; Stoeckert, C.J.; Lazar, M.A. Propagation of adipogenic signals through an epigenomic transition state. *Genes and Development* **2010**, *24*, 1035–1044. doi:10.1101/gad.1907110.
12. Luo, X.; Ryu, K.W.; Kim, D.S.; Nandu, T.; Medina, C.J.; Gupte, R.; Gibson, B.A.; Soccio, R.E.; Yu, Y.; Gupta, R.K.; Kraus, W.L. PARP-1 Controls the Adipogenic Transcriptional Program by PARylating C/EBPβ and Modulating Its Transcriptional Activity. *Molecular Cell* **2017**, *65*, 260–271. doi:10.1016/j.molcel.2016.11.015.
13. Catic, A.; Suh, C.Y.; Hill, C.T.; Daheron, L.; Henkel, T.; Orford, K.W.; Dombkowski, D.M.; Liu, T.; Liu, X.S.; Scadden, D.T. Genome-wide Map of nuclear protein degradation shows NCoR1 turnover as a key to mitochondrial gene regulation. *Cell* **2013**, *155*, 1380–1395. doi:10.1016/j.cell.2013.11.016.
14. Wang, L.; Xu, S.; Lee, J.E.; Baldridge, A.; Grullon, S.; Peng, W.; Ge, K. Histone H3K9 methyltransferase G9a represses PPARγ expression and adipogenesis. *EMBO Journal* **2013**, *32*, 45–59. doi:10.1038/emboj.2012.306.
15. Lim, G.E.; Albrecht, T.; Piske, M.; Sarai, K.; Lee, J.T.; Ramshaw, H.S.; Sinha, S.; Guthridge, M.A.; Acker-Palmer, A.; Lopez, A.F.; Clee, S.M.; Nislow, C.; Johnson, J.D. 14-3-3ζ coordinates adipogenesis of visceral fat. *Nature Communications* **2015**. doi:10.1038/ncomms8671.
16. Chen, X.; Ayala, I.; Shannon, C.; Fourcaudot, M.; Acharya, N.K.; Jenkinson, C.P.; Heikkinen, S.; Norton, L. The diabetes gene and wnt pathway effector TCF7L2 regulates adipocyte development and function. *Diabetes* **2018**, *67*, 554–568. doi:10.2337/db17-0318.

17. Duteil, D.; Metzger, E.; Willmann, D.; Karagianni, P.; Friedrichs, N.; Greschik, H.; Günther, T.; Buettner, R.; Talianidis, I.; Metzger, D.; Schüle, R. LSD1 promotes oxidative metabolism of white adipose tissue. *Nature Communications* **2014**, *5*, 4093. doi:10.1038/ncomms5093.
18. Buchner, D.A.; Charrier, A.; Srinivasan, E.; Wang, L.; Paulsen, M.T.; Ljungman, M.; Bridges, D.; Saltiel, A.R. Zinc finger protein 407 (ZFP407) regulates insulin-stimulated glucose uptake and glucose transporter 4 (Glut4) mRNA. *Journal of Biological Chemistry* **2015**, *290*, 6376–6386. doi:10.1074/jbc.M114.623736.
19. Ryu, K.W.; Nandu, T.; Kim, J.; Challa, S.; DeBerardinis, R.J.; Lee Kraus, W. Metabolic regulation of transcription through compartmentalized NAD+biosynthesis. *Science* **2018**, *360*. doi:10.1126/science.aan5780.
20. Siersbæk, R.; Baek, S.; Rabiee, A.; Nielsen, R.; Traynor, S.; Clark, N.; Sandelin, A.; Jensen, O.N.; Sung, M.H.; Hager, G.L.; Mandrup, S. Molecular architecture of transcription factor hotspots in early adipogenesis. *Cell Reports* **2014**, *7*, 1434–1442. doi:10.1016/j.celrep.2014.04.043.
21. Lo, K.A.; Labadorf, A.; Kennedy, N.J.; Han, M.S.; Yap, Y.S.; Matthews, B.; Xin, X.; Sun, L.; Davis, R.J.; Lodish, H.F.; Fraenkel, E. Analysis of In Vitro Insulin-Resistance Models and Their Physiological Relevance to InVivo Diet-Induced Adipose Insulin Resistance. *Cell Reports* **2013**, *5*, 259–270. doi:10.1016/j.celrep.2013.08.039.
22. Zhao, X.; Yang, Y.; Sun, B.F.; Shi, Y.; Yang, X.; Xiao, W.; Hao, Y.J.; Ping, X.L.; Chen, Y.S.; Wang, W.J.; Jin, K.X.; Wang, X.; Huang, C.M.; Fu, Y.; Ge, X.M.; Song, S.H.; Jeong, H.S.; Yanagisawa, H.; Niu, Y.; Jia, G.F.; Wu, W.; Tong, W.M.; Okamoto, A.; He, C.; Danielsen, J.M.; Wang, X.J.; Yang, Y.G. FTO-dependent demethylation of N6-methyladenosine regulates mRNA splicing and is required for adipogenesis. *Cell Research* **2014**, *24*, 1403–1419. doi:10.1038/cr.2014.151.
23. Brunmeir, R.; Wu, J.; Peng, X.; Kim, S.Y.; Julien, S.G.; Zhang, Q.; Xie, W.; Xu, F. Comparative Transcriptomic and Epigenomic Analyses Reveal New Regulators of Murine Brown Adipogenesis. *PLoS Genetics* **2016**, *12*, e1006474. doi:10.1371/journal.pgen.1006474.
24. Chaudhary, N.; Gonzalez, E.; Chang, S.H.; Geng, F.; Rafii, S.; Altorki, N.K.; McGraw, T.E. Adenovirus Protein E4-ORF1 Activation of PI3 Kinase Reveals Differential Regulation of Downstream Effector Pathways in Adipocytes. *Cell Reports* **2016**, *17*, 3305–3318. doi:10.1016/j.celrep.2016.11.082.
25. Hiraike, Y.; Waki, H.; Yu, J.; Nakamura, M.; Miyake, K.; Nagano, G.; Nakaki, R.; Suzuki, K.; Kobayashi, H.; Yamamoto, S.; Sun, W.; Aoyama, T.; Hirota, Y.; Ohno, H.; Oki, K.; Yoneda, M.; White, A.P.; Tseng, Y.H.; Cypess, A.M.; Larsen, T.J.; Jespersen, N.Z.; Scheele, C.; Tsutsumi, S.; Aburatani, H.; Yamauchi, T.; Kadowaki, T. NFIA co-localizes with PPARγ and transcriptionally controls the brown fat gene program. *Nature Cell Biology* **2017**. doi:10.1038/ncb3590.
26. Abe, Y.; Rozqie, R.; Matsumura, Y.; Kawamura, T.; Nakaki, R.; Tsurutani, Y.; Tanimura-Inagaki, K.; Shiono, A.; Magoori, K.; Nakamura, K.; Ogi, S.; Kajimura, S.; Kimura, H.; Tanaka, T.; Fukami, K.; Osborne, T.F.; Kodama, T.; Aburatani, H.; Inagaki, T.; Sakai, J. JMJD1A is a signal-sensing scaffold that regulates acute chromatin dynamics via SWI/SNF association for thermogenesis. *Nature communications* **2015**, *6*, 7052. doi:10.1038/ncomms8052.
27. Mann, I.K.; Chatterjee, R.; Zhao, J.; He, X.; Weirauch, M.T.; Hughes, T.R.; Vinson, C. CG methylated microarrays identify a novel methylated sequence bound by the CEBPB | ATF4 heterodimer that is active in vivo. *Genome research* **2013**, *23*, 988–97. doi:10.1101/gr.146654.112.
28. Zhou, H.; Kaplan, T.; Li, Y.; Grubisic, I.; Zhang, Z.; Wang, P.J.; Eisen, M.B.; Tjian, R. Dual functions of TAF7L in adipocyte differentiation. *eLife* **2013**, *2*, e00170. doi:10.7554/eLife.00170.
29. Goode, D.K.; Obier, N.; Vijayabaskar, M.S.; Lie-A-Ling, M.; Lilly, A.J.; Hannah, R.; Lichtinger, M.; Batta, K.; Florkowska, M.; Patel, R.; Challinor, M.; Wallace, K.; Gilmour, J.; Assi, S.A.; Cauchy, P.; Hoogenkamp, M.; Westhead, D.R.; Lacaud, G.; Kouskoff, V.; Göttgens, B.; Bonifer, C. Dynamic Gene Regulatory Networks Drive Hematopoietic Specification and Differentiation. *Developmental cell* **2016**, *36*, 572–87. doi:10.1016/j.devcel.2016.01.024.
30. Menéndez-Gutiérrez, M.P.; Rőszer, T.; Fuentes, L.; Núñez, V.; Escolano, A.; Redondo, J.M.; De Clerck, N.; Metzger, D.; Valledor, A.F.; Ricote, M. Retinoid X receptors orchestrate osteoclast differentiation and postnatal bone remodeling. *The Journal of clinical investigation* **2015**, *125*, 809–23. doi:10.1172/JCI77186.
31. Soccio, R.E.; Li, Z.; Chen, E.R.; Foong, Y.H.; Benson, K.K.; Dispirito, J.R.; Mullican, S.E.; Emmett, M.J.; Briggs, E.R.; Peed, L.C.; Dzeng, R.K.; Medina, C.J.; Jolivert, J.F.; Kissig, M.; Rajapurkar, S.R.; Damle, M.;

Lim, H.W.; Won, K.J.; Seale, P.; Steger, D.J.; Lazar, M.A. Targeting PPARγ in the epigenome rescues genetic metabolic defects in mice. *The Journal of clinical investigation* **2017**, *127*, 1451–1462. doi:10.1172/JCI91211.

32. Hamza, M.S.; Pott, S.; Vega, V.B.; Thomsen, J.S.; Kandhadayar, G.S.; Ng, P.W.P.; Chiu, K.P.; Pettersson, S.; Wei, C.L.; Ruan, Y.; Liu, E.T. De-novo identification of PPARgamma/RXR binding sites and direct targets during adipogenesis. *PloS one* **2009**, *4*, e4907. doi:10.1371/journal.pone.0004907.
